# Supplementary material for: Content-rich biological network constructed by mining PubMed abstracts
Source: BMC Bioinformatics. 2004 Oct 8;5:147. doi: 10.1186/1471-2105-5-147 (PMC528731; doi:10.1186/1471-2105-5-147)
Supplement: Additional File 2 — The original results of the above study (non-essential files are deleted to keep the file size under the limit set by BMC bioinformatics). [file 1471-2105-5-147-S2.bz2 › chilibotAdditionalFile2/dip10/498ID10187774E10661/html/TGFB3_ACVRL1.html]

 


 **TGFB3** and **ACVRL1** 
  
Found 14 abstracts in PubMed, retrieved 10.  
 

 What does Google say? 
 PDF only 
| .edu only 

---

**Interactive relationship** (e.g. stimulation, inhibition, etc)

**Non-interactive relationship** (e.g. studied together, co-existance, homology, etc.)

- We show that TGF beta1 and TGF beta3  [ **TGFB3** ] , as well as a third unknown ligand present in serum, can activate chimeric ALK 1  [ **ACVRL1** ] .  Ref: 10187774 J Biol Chem, 1999
- We therefore studied the expression of UPA, UPAR, IGF2R, ALK5, TGFBR2, TGFBR3, ENG, ALK1  [ **ACVRL1** ] , TGFB1, TGFB2, and  **TGFB3**  in a series of 14 pancreatic carcinoma cell lines.  Ref: 11408925 Int J Oncol, 2001
- METHODS AND RESULTS The spatial relationships between TGF beta1, TGF beta3  [ **TGFB3** ] , ALK  [ **ACVRL1** ]  5, and TbetaR II expression were compared in aortic segments from 21 subjects.  Ref: 10359732 Circulation, 1999
- Boston minipigs, treated with tranilast or vehicle, were subjected to endoluminal stenting, and the expression of TGF beta1 and TGF beta3  [ **TGFB3** ] , the expression of their signaling receptors ALK  [ **ACVRL1** ]  5 and TbetaR II, leukocyte numbers around the stent struts, and neointima development were assessed over 28 days.  Ref: 12067902 Arterioscler Thromb Vasc Biol, 2002
